# Peritoneal Mouse as Detected on ^18^F-FDG PET-CT

**DOI:** 10.3389/fonc.2013.00083

**Published:** 2013-04-10

**Authors:** Talha Allam, Razi Muzaffar, Nghi C. Nguyen, Medhat M. Osman

**Affiliations:** ^1^Department of Radiology, Saint Louis UniversitySaint Louis, MO, USA; ^2^Division of Nuclear Medicine, Department of Radiology, Saint Louis UniversitySaint Louis, MO, USA; ^3^Division of Nuclear Medicine, Department of Radiology, Saint Louis VA Medical CenterSaint Louis, MO, USA

**Keywords:** peritoneal mouse, loose body, epiploic appendage, PET-CT, colon cancer

## Abstract

We present the case of a 77-year-old male with a history of prostate cancer. Follow up PET-CT and contrast-enhanced CT demonstrated a small peritoneal loose body or “mouse” in the pelvis. This is an uncommon, benign, asymptomatic finding which is usually incidentally discovered. The significance of being aware of this entity is to distinguish it from metastasis, especially in patients with known abdominal and pelvic malignancies.

## Case Presentation

**Figure 1:**
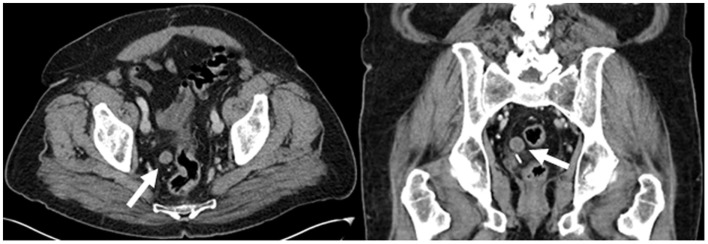
**Contrast-enhanced CT demonstrates a well circumscribed round soft tissue density measuring 1.7 cm in the right hemipelvis adjacent to the sigmoid colon (white arrows)**.

**Figure 2:**
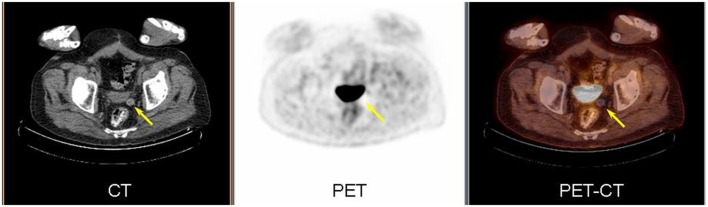
**PET/CT images demonstrate the soft tissue lesion has moved from the right to left hemipelvis (arrow) on low dose CT (left)**. The lesion demonstrated no metabolic activity on PET (center) and fused image (right).

A 77-year-old male with a remote history of prostate cancer was referred to our imaging facility after undergoing a prostatectomy at an outside facility. He presented to the emergency department with non-specific abdominal pain. A contrast-enhanced CT abdomen and pelvis was performed which showed no acute findings to explain his symptoms. However, a well circumscribed round soft tissue density measuring 1.7 cm was seen in the right hemipelvis adjacent to the sigmoid colon (Figure 1). The lesion was not contiguous with bowel. Given the patient’s history of prostate cancer, a peritoneal metastasis or necrotic lymph node was suggested. On the coronal image, a surgical clip inferior to the lesion was from prior prostatectomy. ^18^F-FDG PET-CT was performed 3 days following the contrast-enhanced CT. The low dose CT showed that the lesion had moved from the right to the left and was located near the urinary bladder (Figure 2). The lesion demonstrated no FDG uptake on PET. Based on the mobility of the lesion, a diagnosis of a peritoneal loose body or “mouse” was made.

## Background

Peritoneal loose bodies or “mice” are commonly small mobile lesions falling into the category of an incidentaloma. They are found within the peritoneum during surgery or radiographically and have little clinical significance. However, in patients with a history of malignancy they can lead to altered patient management if perceived as a site of metastasis. We report a case of a peritoneal mouse in a patient with prostate cancer and differentiating it from a metastatic lesion.

## Discussion

Peritoneal mice have not been previously reported in nuclear medicine literature. There are a few surgical case reports describing this uncommon entity (Nomura et al., 2003; Asabe et al., 2005; Mohri et al., 2007; Kavanagh et al., 2010). Two radiology case reports have described this finding on CT (Ohgitani et al., 2004; Gayer and Petrovitch, 2011). The most commonly accepted hypothesis suggests that peritoneal mice arise from the colonic epiploic appendages. Epiploic appendages are fat containing structures along the antimesenteric tenia of the colon. An epiploic appendage may torse on its axis resulting in infarction and fat necrosis. In the acute setting, the torsion may present as acute abdominal pain and could mimic appendicitis. In cases of chronic torsion, ischemia leads to saponification and calcification of the fat contents and atrophy of the pedicle (Kavanagh et al., 2010). The epiploic appendage detaches from the colon and becomes a peritoneal mouse. Since they are avascular and no longer connected to bowel, they can freely move in the peritoneum as loose bodies. On CT a peritoneal mouse manifests as a well circumscribed round soft tissue density which may have thick or calcified rims. The most common location is adjacent to the colon, particularly the sigmoid colon. It does not enhance with contrast and shows no FDG uptake on PET. Most peritoneal mice are asymptomatic incidentalomas which are usually 0.5–2.5 cm in diameter. Rare giant peritoneal mice measuring 5–10 cm in diameter may cause symptoms due to mass effect. Cases of urinary and intestinal obstruction have been reported due to giant peritoneal mice (Bhandarwar et al., 1996; Ghosh et al., 2006). A case of a peritoneal mouse mimicking a rectal leiomyoma has been reported (Takada et al., 1998).

## Conclusion

A peritoneal loose body or mouse is a benign finding. The significance of being aware of this entity is to distinguish it from metastasis, especially in patients with known abdominal or pelvic malignancies.

## Conflict of Interest Statement

The authors declare that the research was conducted in the absence of any commercial or financial relationships that could be construed as a potential conflict of interest.
